# A temporally harmonised gridded building-stock dataset for the Global South, 2016-2023

**DOI:** 10.12688/gatesopenres.16394.1

**Published:** 2026-07-30

**Authors:** Wen-Bin Zhang, Tom McKeen, Rhorom Priyatikanto, Weixuan Fang, Natalia Tejedor-Garavito, Alessandro Sorichetta, Andrew J. Tatem, Maksym Bondarenko

**Affiliations:** 1 University of Southampton School of Geography and Environmental Science, Southampton, England, UK; 2 Universita degli Studi di Milano Dipartimento di Scienze della Terra Ardito Desio, Milan, Lombardy, Italy

**Keywords:** Global South; Gridded Building footprints; Google Open Buildings; settlement change

## Abstract

**Background:**

Rapid settlement change across the Global South is transforming the built environment, yet consistent spatio-temporal information on buildings remains limited in many low- and middle-income countries. Multi-temporal building datasets derived from satellite imagery can contain year-to-year inconsistencies caused by variation in image availability, acquisition conditions, and model performance, limiting their use for longitudinal analysis.

**Methods:**

We developed a spatio-temporally harmonised gridded building-characteristics dataset for 130 countries in the Global South covering 2016–2023. The dataset is derived from annual gridded layers aggregated from the Google Open Buildings 2.5D Temporal dataset at a spatial resolution of 3 arc-seconds, approximately 100 metres at the equator. For each grid cell and year, three building metrics are provided: building count, building surface area, and building volume. To improve temporal consistency, we developed a deterministic adaptive forward-backward temporal smoother inspired by Kalman updating principles, rather than a formal Kalman filter or state-space model. The adaptive gain assigns less weight to annual observations that depart strongly from a grid cell’s multi-year trajectory relative to a spatially smoothed baseline deviation measure.

**Results:**

Technical validation shows that the harmonisation reduces implausible year-to-year oscillations and relative zigzag amplitudes across the three building metrics. At the same time, high rank correlations between raw and harmonised annual layers indicate that the procedure preserves the broad spatial distribution of building characteristics. City-level examples further illustrate how the method handles intermittent missing observations, abrupt annual changes, and short-term oscillations while retaining longer-term temporal trajectories.

**Conclusions:**

The resulting dataset provides a temporally consistent multi-year representation of building characteristics across data-scarce regions of the Global South. It is designed to support analyses of settlement expansion, population distribution, infrastructure planning, and environmental risk, while remaining best suited to multi-year trends rather than detection of short-term individual events.

## 1. Introduction

Settlements in the Global South are undergoing rapid and heterogeneous transformation. Urban populations are growing, built-up areas are expanding and densifying, and new infrastructure is being added at a pace that often outstrips the capacity of statistical and planning systems to monitor these changes ( UN DESA Population Division, 2025; UN Statistics Division, 2024). Similar pressures are also evident in rural areas, peri-urban settlements, and small towns, particularly where fertility remains high and settlement growth is weakly captured by formal systems ( Lerch, 2019). However, spatially explicit and temporally consistent information on building location, density, and change remains limited in many low- and middle-income countries ( Biljecki et al., 2021; Prieto-Curiel et al., 2023). Conventional data sources, including censuses, administrative registers, cadastres, and in situ surveys, are often infrequent, unevenly updated, and not designed to provide detailed geospatial information on building form and dynamics ( UN Statistics Division, 2017). They may also be incomplete in informal settlements and peri-urban areas, where urban expansion is often most rapid ( Kraff et al., 2020). As a result, governments, development agencies, and researchers face persistent difficulties in quantifying urbanisation trajectories, assessing infrastructure gaps, estimating population exposure to hazards, and monitoring progress towards the Sustainable Development Goals.

Over the past decade, advances in Earth observation and machine learning have begun to transform the mapping of the built environment ( Zhu et al., 2017). Global and regional building footprint datasets derived from satellite imagery now map billions of structures at increasingly fine spatial resolutions ( Sirko et al., 2021; Milojevic-Dupont et al., 2023; Microsoft, 2026). Products such as Microsoft Global Building Footprints, OpenStreetMap-based footprints, Ecopia AI datasets, and global built-up area layers such as the Global Human Settlement Layer provide unprecedented spatial detail and coverage ( Haklay & Weber, 2008; Chamberlain et al., 2024; Corbane et al., 2020; Corbane et al., 2021). More recent aggregation and harmonisation initiatives, including Overture Maps and the GlobalBuildingAtlas, further consolidate multiple building-footprint sources and extend global coverage towards consistent 2D and 3D building representations ( Zhu et al., 2025). Complementary attribute-enrichment efforts, such as GHS-OBAT, have also begun to attach information on building height, construction epoch, functional use, and compactness to global footprint datasets ( Florio et al., 2025). Combined with global population grids and other geospatial covariates, these datasets have enabled new analyses of settlement patterns, urban form, infrastructure access, and exposure to environmental risks, particularly in regions where official statistics are limited ( Tatem, 2017). Despite these advances, most existing building datasets provide static representations of the built environment or are updated only intermittently ( Marconcini et al., 2020; Chamberlain et al., 2024; Minghini et al., 2024). As a result, they are poorly suited to studying how settlements evolve over time. Analyses of urban expansion, densification, infrastructure development, and settlement growth require consistent multi-year observations. Producing such time series is challenging because satellite-derived building detection is sensitive to changes in observation conditions, including cloud cover, atmospheric conditions, seasonal vegetation, and viewing geometry ( Chen et al., 2004; Jönsson & Eklundh, 2004). Differences in image availability, preprocessing pipelines, or model versions between years can introduce artificial fluctuations that do not correspond to real changes in the built environment ( Qiu et al., 2019; Frantz et al., 2023). While such artefacts may be acceptable for single-year mapping, they become problematic for longitudinal analyses that aim to quantify growth rates, identify inflection points in urban expansion, or evaluate the impacts of policies and shocks.

The Google Open Buildings project represents one of the first large-scale efforts to provide explicitly multi-temporal building information for the Global South ( Sirko et al., 2023). Using deep learning applied to multispectral imagery from the Sentinel-2 mission, the Google Open Buildings 2.5D Temporal dataset provides annual maps of building presence, count, and height from 2016 onwards across large parts of Africa and other regions ( Sirko et al., 2023). By aggregating building-level detections to regular spatial grids, these data can be transformed into building characteristics such as building counts, total footprint area, perimeter, and volume ( Priyatikanto et al., 2025). Such gridded building metrics provide valuable covariates for population modelling, exposure assessment, urban growth analysis, and infrastructure planning ( Rubinyi et al., 2021; Boo et al., 2022; Lochhead et al., 2026). However, the use of multi-temporal building data derived from satellite imagery also introduces important challenges related to temporal consistency. Analyses of urban expansion, densification, infrastructure development, and settlement growth require consistent multi-year observations. Producing such time series is challenging because satellite-derived building detection is sensitive to changes in observation conditions, including cloud cover, atmospheric conditions, seasonal vegetation, and viewing geometry ( Chen et al., 2004; Jönsson & Eklundh, 2004). Differences in image availability, preprocessing pipelines, or model versions between years can introduce artificial fluctuations that do not correspond to real changes in the built environment ( Qiu et al., 2019; Frantz et al., 2023). While such artefacts may be acceptable for single-year mapping, they become problematic for longitudinal analyses that aim to quantify growth rates, identify inflection points in urban expansion, or evaluate the impacts of policies and shocks.

In other domains of Earth observation, similar issues have been addressed through temporal smoothing and harmonisation techniques such as Kalman filtering, state-space models, and other time-series regularisation approaches ( Kalman, 1960; Rauch et al., 1965; Durbin & Koopman, 2012). For example, TIMESAT combines adaptive Savitzky–Golay filtering with fitted asymmetric Gaussian and double-logistic functions to reduce noise and extract seasonal characteristics from comparatively dense vegetation-index time series ( Jönsson & Eklundh, 2004). In that context, asymmetry describes the shape of a fitted seasonal curve, whereas in the present procedure it refers to the direction-specific treatment of unusually large increases and decreases. Such methods have also contributed to the production of temporally coherent vegetation and climate products by reducing irregular variation and filling observational gaps while preserving broader trends ( Chen et al., 2004; Hersbach et al., 2020). Annual building-characteristic series nevertheless present a distinct harmonisation problem. They are short and non-seasonal and may contain persistent increases, genuine abrupt losses, and directionally asymmetric detection errors.

In this study, we present a spatio-temporally harmonised dataset of annual building characteristics for the Global South covering the period 2016–2023. Starting from annual gridded products ( Priyatikanto et al., 2025) derived from Google Open Buildings 2.5D Temporal dataset, we construct time series of three building metrics for each grid cell, including building count, surface area, and building volume. These metrics capture complementary aspects of the built environment, from the number of structures to horizontal built-up extent and vertical building mass, and are widely relevant to population distribution modelling, socio-economic analysis, and assessments of built-environment microclimate ( Nieves et al., 2021; Han et al., 2022; Liu et al., 2023; Zhu et al., 2025). To improve temporal consistency, we developed a deterministic adaptive forward-backward smoother inspired by the recursive updating principles of Kalman filtering. The procedure combines direction-specific cap adjustment with an adaptive smoothing strength that varies among grid cells and years according to locally estimated temporal deviation and a spatially regularised baseline. In contrast to seasonal curve-fitting methods, the approach is designed for short annual building-characteristic series and seeks to reduce isolated fluctuations while retaining persistent multi-year changes. Temporary gap filling enables temporal processing, after which the leading missing segment before the first valid observation is restored to NA. The resulting dataset provides a reproducible, temporally stabilised representation of annual building characteristics for applications requiring consistent multi-year information across data-scarce regions.

## 2. Input data and spatial framework

**Figure 1.**
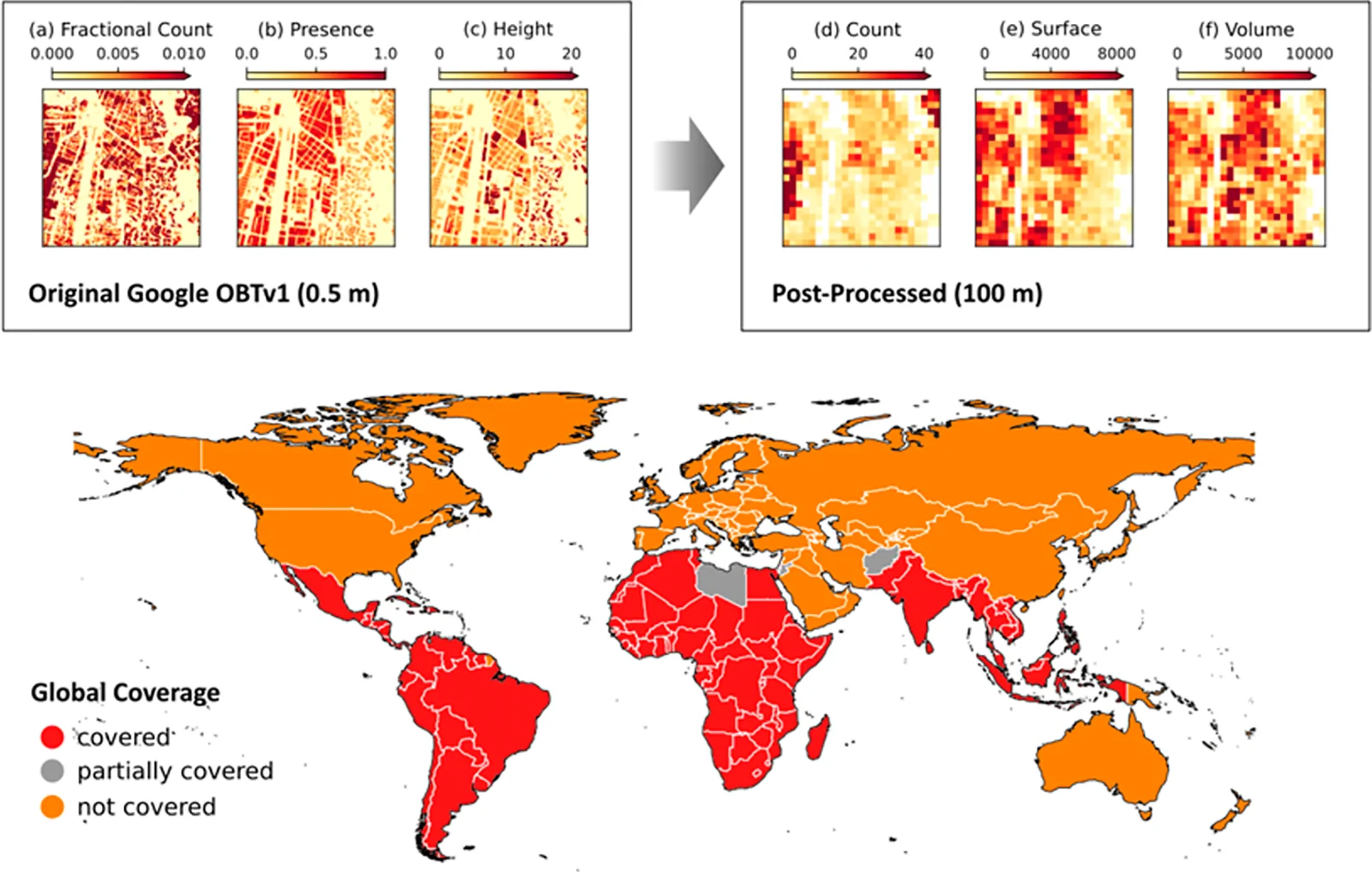
Illustration of the Google Open Buildings 2.5D Temporal v1 (OBTv1) dataset (a, b, c) and the corresponding building metrics derived at 100 m resolution (d, e, f ) and the associated spatial coverage.

This dataset builds on a previously released 3 arc-seconds (approximately 100 m at the equator) gridded building-characteristics product ( Priyatikanto et al., 2025) for the Global South, which aggregates annual detections from Google Open Buildings 2.5D Temporal dataset V1 ( Sirko et al., 2023) to the WorldPop master grid ( Woods et al., 2025) in EPSG:4326. The dataset is available for 130 countries across Africa, South Asia, South-East Asia, Latin America and the Caribbean. These countries were included because they constitute the geographic coverage of the source product for which annual gridded building-characteristic layers are available throughout the 2016–2023 study period. Thus, their inclusion was determined by the availability and spatial coverage of the input data rather than by an independent sampling or country-selection procedure. In the source product, building-level information derived from Sentinel-2 imagery is summarised into annual gridded layers for the period 2016–2023. Three annual building metrics are processed for each grid cell, including 1) building count, 2) building surface area, and 3) building volume, with volume defined as the sum of footprint area multiplied by estimated building height. These layers are derived through a combination of arithmetic operations and spatial aggregation ( Priyatikanto et al., 2025). Building count is computed by integrating fractional building detections within each grid cell. Total footprint area is obtained by summing 50 cm pixels where building presence exceeds a specified confidence threshold. Building volume is calculated by aggregating the product of building footprint and corresponding height estimates within each grid cell. An overview of the dataset coverage and the derivation of these metrics at 100 m resolution is illustrated in Figure 1.

**Figure 2.**
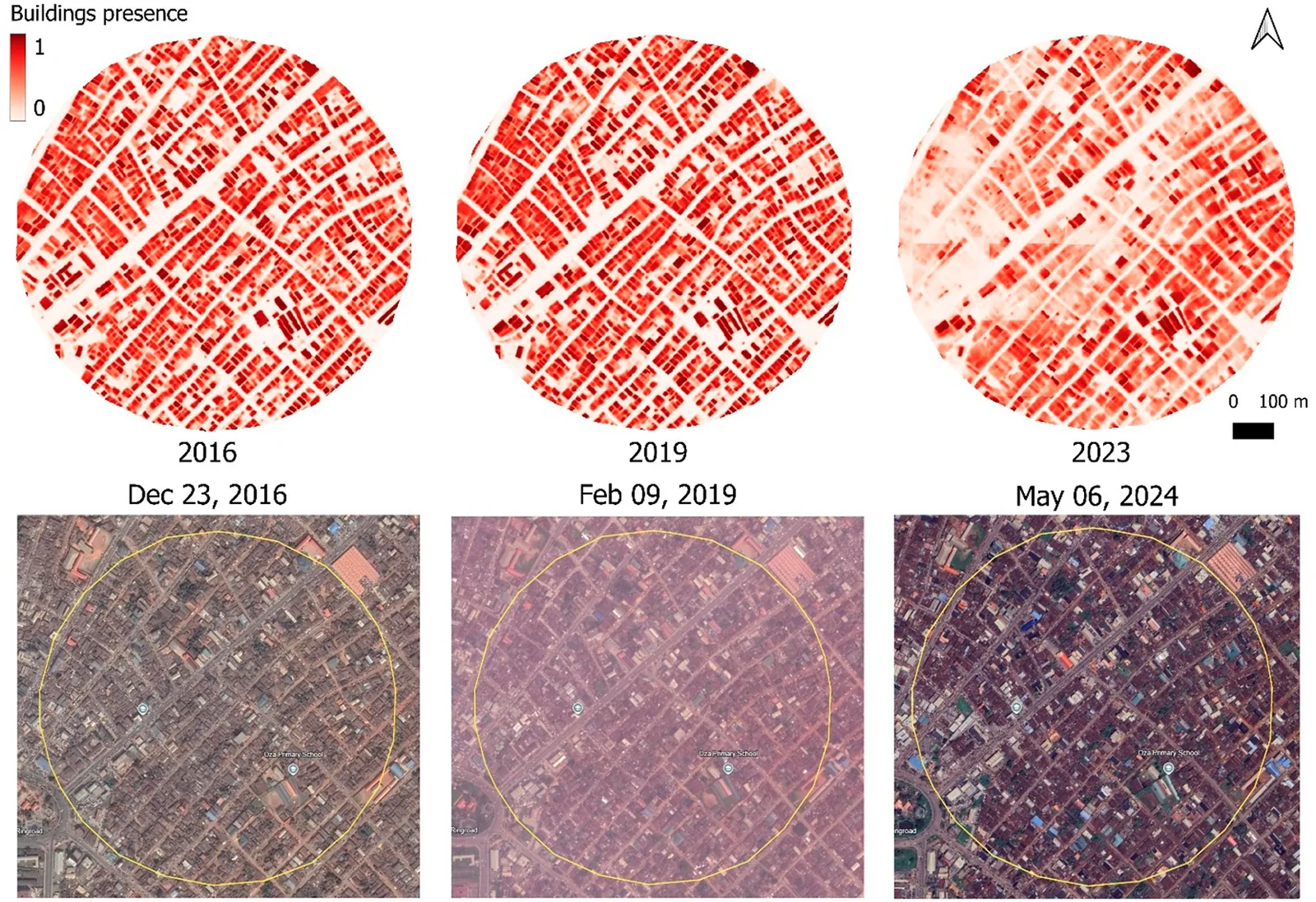
Comparison of gridded building presence layer from the Google Open Buildings 2.5D Temporal dataset and Google Earth historical imagery (2016, 2019, 2023). The area of interest is indicated by a circle centred at 6.335356°N, 5.627852°E.

In this study, these annual gridded layers were used as input time series for additional spatio-temporal harmonisation. The objective of this second-stage processing was to reduce implausible year-to-year fluctuations while preserving broad temporal trajectories and spatial structure. As illustrated in Figure 2, these fluctuations are primarily linked to variations in image availability, acquisition conditions, and model performance, which can propagate through the aggregation process and generate inconsistencies in derived building metrics such as count, surface area, and volume. The resulting harmonised gridded dataset differs from the original product both in numerical values and in intended use. Whereas the original dataset represents a direct annual aggregation of building detections, the harmonised dataset is specifically designed for longitudinal analyses that require temporally consistent grid-cell estimates. All outputs retain the spatial properties of the input dataset, including grid alignment, spatial resolution, extent, and coordinate reference system.

## 3. Methodology

The harmonisation workflow comprises a sequence of operations, including 1) preparation of the annual input layers, 2) temporary filling of missing values to enable temporal processing, 3) asymmetric cap adjustment, 4) adaptive forward and backward smoothing, 5) time-varying blending of the directional estimates, 6) restoration of the leading missing segment before the first valid observation, and 7) export of the harmonised annual GeoTIFF layers. Each operation is described in the following subsections.

### 3.1. Temporal gap filling for smoothing

The annual building-characteristic time series contain missing values (NA) for specific grid cells and years owing to gaps in the input satellite imagery or failures in the building-detection pipeline. These missing values are distinct from recorded zeros; an NA indicates that no valid estimate was available, whereas zero indicates that the source data reported no detected building count, surface area, or volume. Nevertheless, a recorded zero does not necessarily confirm the true absence of buildings because it may also reflect non-detection.

Because the temporal smoothing procedure requires a complete time series at each location, missing values were temporarily filled before filtering. Let r(i,t) denote the raw value of a building metric for grid cell i and year t , where t∈{2016,…,2023} . The resulting complete sequence, $\tilde{r}\left(i,t\right)$ , was defined as

$$\tilde{r}\left(i,t\right)=\left\{ \begin{array}{cc} r\left(i,t\right), & \text{if}\;r\left(i,t\right)\;\text{is observed},\\ 0, & \text{if}\;r\left(i,t\right)\;\text{is}\;\mathrm{NA}. \end{array}\right.$$ (1)

This temporary imputation allows the sequence to represent the emergence of detected buildings and apparent reductions in detected building stock. It is used only to enable the smoothing algorithm and does not alter the underlying raw data. After harmonisation, cells without any valid observations remain entirely NA, and years preceding the first valid observation are reset to NA. Internal and trailing missing years occurring after the first valid observation are not re-masked and therefore retain harmonised gap-filled values. Consequently, an NA in the final dataset represents either a cell with no valid observations or a year preceding the first valid observation.

### 3.2. Asymmetric cap adjustment of implausible annual changes

The raw annual building-metric series may exhibit abrupt year-to-year jumps that are unlikely to reflect real settlement dynamics and are more plausibly caused by variation in satellite imagery quality, observation gaps, or instability in the building-detection pipeline. To reduce such artefacts while preserving plausible expansion trajectories, an asymmetric cap adjustment is applied to each grid-cell time series prior to temporal smoothing. For each grid cell, the anchor year $t_{i}^{*}$ is defined as the year with the highest observed value in the gap filling time series, $t_{i}^{*}=\arg\max_{t}\tilde{r}\left(i,t\right)$ . This choice reflects the assumption that the maximum observed value is most likely to represent the best-quality estimate or say a full capture of built presence for that cell, whereas unusually low values are more likely to arise from omission errors. An asymmetric correction rule is then applied outward from the anchor year. The rule differs for years before and after $t_{i}^{*}$ .

For years before the anchor year, smaller values are permitted without restriction, since lower historical values are consistent with settlement growth over time. However, if an earlier observation is implausibly large relative to the adjusted value in the adjacent later year, it is capped downward. Denoting the adjusted series by $\bar{r}\left(i,t\right)$ , the upper bound for year t before the anchor year is defined from the adjusted value at the nearest subsequent observed year t+1 as

$$U\left(i,t\right)=\left\{ \begin{array}{ll} \bar{r}\left(i,t+1\right)-3, & \text{for building count},\\ \bar{r}\left(i,t+1\right)-500, & \text{for building surface area}\;\left(m^{2}\right),\\ \bar{r}\left(i,t+1\right)-1500, & \text{for building volume}\;\left(m^{3}\right). \end{array}\right.$$ (2)

If $\tilde{r}\left(i,t\right)>U\left(i,t\right)$ , then the adjusted value is set to the cap, $\bar{r}\left(i,t\right)=U\left(i,t\right),$ otherwise $\bar{r}\left(i,t\right)=\tilde{r}\left(i,t\right)$ . The thresholds used to constrain annual change were set to 3 buildings, 500 m ^2^ of building surface area, and 1500 m ^3^ of building volume. These values are close to the 10th percentile of non-zero grid-cell values for the corresponding metrics and were selected as conservative upper bounds on plausible year-to-year change. This limits unrealistically large annual gains or losses at the grid-cell level while preserving smaller changes that may reflect genuine building emergence, removal, or modification.

For years after the anchor year, all values are smaller compared to the anchor year by definition. However, if a later observation is implausibly small relative to the adjusted value in the adjacent earlier year, it is raised to a lower bound, which is defined as

$$L\left(i,t\right)=\left\{ \begin{array}{ll} \max\left\{\bar{r}\left(i,t-1\right)-3,0\right\}, & \text{for building count},\\ \bar{r}\left(i,t-1\right)-500, & \text{for building surface area}\;\left(m^{2}\right),\\ \bar{r}\left(i,t-1\right)-1500, & \text{for building volume}\;\left(m^{3}\right). \end{array}\right.$$ (3)

If $\tilde{r}\left(i,t\right)<L\left(i,t\right)$ , then $\bar{r}\left(i,t\right)=L\left(i,t\right),$ otherwise $\bar{r}\left(i,t\right)=\tilde{r}\left(i,t\right)$ .

This procedure is applied sequentially outward from the anchor year, using the adjusted value from the adjacent year as the reference for the next step. In this way, the adjustment propagates gradually through the time series rather than imposing a fixed constraint relative to the anchor year alone. The adjustment is intentionally asymmetric. It suppresses implausibly high pre-anchor values and implausibly low post-anchor values, while retaining plausible monotonic growth. This design avoids over-correcting rapidly developing locations, where large increases through time may be real. The resulting adjusted series $\bar{r}\left(i,t\right)$ is then used as the input for subsequent spatio-temporal smoothing.

### 3.3. Adaptive forward-backward temporal smoothing

**3.3.1. Temporal-deviation measures and spatial regularisation baseline**

We applied a deterministic adaptive forward–backward temporal smoother independently to each grid cell and building metric. The procedure is inspired by the recursive update structure of Kalman filtering, but it is not a formal Kalman filter or state-space model. In particular, it does not specify probability distributions for a latent building-stock state, observation error, or process error, and it does not propagate posterior uncertainty. The quantities Q(i,t) and $Q_{\text{base}}\left(i\right)$ are therefore interpreted as heuristic temporal-deviation measures used to control smoothing strength, rather than as statistically estimated error variances.

The pre-processed series $\bar{r}\left(i,t\right)$ is treated as the observed input time series for each grid cell i . To characterise temporal change, annual differences are first computed as $D\left(i,t\right)=\bar{r}\left(i,t\right)-\bar{r}\left(i,t-1\right),t=2017,\ldots ,2023$ , and a baseline linear increment across the full observation period is defined as $D_{\text{base}}\left(i\right)=\left(\bar{r}\left(i,2023\right)-\bar{r}\left(i,2016\right)\right)/7$ . The annual differences describe local year-to-year change, while the baseline increment approximates the mean annual change over the entire time span.

A deviation proxy Q(i,t) is then defined to measure the degree to which a given annual increment deviates from this baseline trend. For the first and last years, $Q\left(i,2016\right)={\left[D\left(i,2017\right)-D_{\text{base}}\left(i\right)\right]}^{2}$ and $Q\left(i,2023\right)={\left[D\left(i,2023\right)-D_{\text{base}}\left(i\right)\right]}^{2}.$ For intermediate years, the deviation proxy is computed as the average of the squared deviations of the adjacent increments:

$$Q\left(i,t\right)=[D\left(i,t\right)-D_{\text{base}}\left(i\right)]{}^{2}+[D(i,t+1)-D_{\text{base}}\left(i\right)]{}^{2}/2,t=2017,\ldots ,2022.$$ (4)

This quantity represents a local estimate of temporal variability at each grid cell and year.

To stabilise the smoothing procedure and promote spatial consistency, a spatially smooth baseline variance field $Q_{\text{base}}\left(i\right)$ is constructed. First, the minimum value of Q(i,t) across all years is computed for each grid cell. This minimum is used as a conservative estimate of the typical variance at that location. The resulting raster is then spatially smoothed using a 3×3 Gaussian kernel with standard deviation σ=0.8 , normalised to sum to one, applied through focal convolution. This produces a spatially continuous baseline variance field that varies smoothly across space but remains constant over time.

**3.3.2. Forward–backward temporal smoothing**

Temporal smoothing was performed using a forward–backward Kalman-inspired filtering procedure developed in this study and applied independently to each grid cell and building metric. The method draws on the general principles of Kalman filtering and smoothing ( Kalman, 1960; Pnevmatikakis et al., 2014), but was adapted to the specific problem of reducing implausible year-to-year fluctuations in gridded building metrics. Spatial variation in smoothing strength is introduced through the baseline variance field $Q_{\text{base}}\left(i\right)$ .

In the forward pass, the smoothed series is initialised at the first year as ${\hat{r}}_{\mathrm{fwd}}\left(i,2016\right)=\bar{r}\left(i,2016\right)$ . The estimate is then updated sequentially for t=2017,…,2023 . At each step, an adaptive gain factor is computed as $K\left(i,t\right)=Q_{\text{base}}\left(i\right)/\left(Q_{\text{base}}\left(i\right)+Q\left(i,t\right)+\varepsilon \right)$ . For numerical stability, a small constant $\varepsilon =10^{-6}$ is added to denominators in gain calculations. The adaptive gain satisfies 0≤K(i,t)<1 and determines how strongly the current adjusted observation influences the updated estimate. When Q(i,t) is small relative to $Q_{\text{base}}\left(i\right)$ , the annual change is close to the grid cell’s multi-year trajectory and K(i,t) approaches one. The smoother therefore assigns greater weight to the current observation. Conversely, when Q(i,t) is large relative to $Q_{\text{base}}\left(i\right)$ , the annual increment departs strongly from the baseline trajectory, K(i,t) approaches zero, and the update relies more heavily on the preceding forward or backward estimate. Thus, the gain acts as a deterministic, data-adaptive regularisation weight rather than as a conventional Kalman gain derived from estimated process and observation-error covariance matrices.

The procedure relies on several assumptions. First, isolated annual increases or decreases that depart strongly from the multi-year trajectory are assumed to be more likely to reflect incomplete observations or detection instability than persistent changes in building stock. Second, changes that persist across several consecutive years are treated as more credible than single-year reversals. Third, the asymmetric cap assumes that omission-related underestimation is more common than large false-positive overestimation and that the maximum observed value provides a useful anchor for the locally observed building stock. Fourth, the spatial regularisation of $Q_{\text{base}}$ assumes that neighbouring grid cells have partly related baseline detection variability. These assumptions are intended to improve temporal consistency but do not establish whether any individual annual change is genuine.

The forward estimate is then updated according to

$${\hat{r}}_{\mathrm{fwd}}\left(i,t\right)={\hat{r}}_{\mathrm{fwd}}\left(i,t-1\right)+K\left(i,t\right)\left(\bar{r}\left(i,t\right)-{\hat{r}}_{\mathrm{fwd}}\left(i,t-1\right)\right).$$ (5)

This update combines the previous estimate with the current observation, with weights determined by the relative magnitude of the baseline variance and the local deviation proxy. A backward pass is subsequently applied to incorporate information from later years. The backward estimate is initialised at the final year, ${\hat{r}}_{\mathrm{bwd}}\left(i,2023\right)=\bar{r}\left(i,2023\right)$ , and updated recursively for t=2022,…,2016 :

$${\hat{r}}_{\mathrm{bwd}}\left(i,t\right)={\hat{r}}_{\mathrm{bwd}}\left(i,t+1\right)+K\left(i,t\right)\left(\bar{r}\left(i,t\right)-{\hat{r}}_{\mathrm{bwd}}\left(i,t+1\right)\right).$$ (6)

Finally, the forward and backward estimates are combined using a time-varying weighted blend to obtain the harmonised time series,

$$\hat{r}\left(i,t\right)=\omega_{\mathrm{fwd}}\left(i,t\right){\hat{r}}_{\mathrm{fwd}}\left(i,t\right)+\omega_{\mathrm{bwd}}\left(i,t\right){\hat{r}}_{\mathrm{bwd}}\left(i,t\right),\;t=2016,\ldots ,2023,$$ (7)

where $\omega_{\mathrm{fwd}}\left(i,t\right)=1-\omega_{\mathrm{bwd}}\left(i,t\right)$ . The blending weights are defined separately for each grid cell according to the temporal span of valid observations. For a given cell, the first year with a valid observation is assigned a forward weight of 0.9 and the last year a forward weight of 0.1. Weights for intermediate years are obtained by linear interpolation between these endpoints, while backward weights are defined as the complement. For years outside the observed temporal span, weights are set equal to the nearest endpoint value, such that years preceding the first observation retain a forward weight of 0.9 and years following the last observation retain a forward weight of 0.1. This formulation assigns greater influence to the forward estimate at the beginning of the time series and to the backward estimate at the end, while balancing the two contributions in intermediate years. This dynamic weighting reduces sensitivity to the temporal direction of filtering and avoids imposing arbitrary equal weighting in years lacking direct observations, while maintaining temporal consistency within each grid cell.

Grid cells with fewer than three valid annual observations were treated explicitly. Cells with no valid observations were retained as entirely missing and were excluded from temporal processing. For cells with only one valid observation, forward–backward smoothing was not applied because neither a temporal trajectory nor distinct first- and last-observation blending weights could be defined reliably; the single observed value was retained and all other years remained NA. Cells with two valid observations were processed using the standard procedure. The earlier and later valid years were assigned forward weights of 0.9 and 0.1, respectively, and weights for intervening years were obtained by linear interpolation. Years preceding the first valid observation were subsequently restored to NA in accordance with the general missing-data rule. This explicit treatment prevents a single observation from being propagated into an unsupported temporal trajectory.

After temporal smoothing, only the original leading missing segment is restored, i.e., all years preceding the first valid observation in the raw series are reset to NA. Trailing and internal gaps are retained as smoothed values in the final harmonised series. This preserves the original absence of observations before first appearance while maintaining temporal continuity thereafter. Overall, the method does not explicitly classify individual observations as detection artefacts or genuine building-stock changes. Instead, it down-weights observations according to their consistency with the local multi-year trajectory and spatially regularised deviation baseline. Persistent changes can therefore be retained, whereas isolated abrupt changes are more strongly attenuated. This design may also smooth genuine abrupt events, including rapid construction, demolition, disaster damage, or redevelopment. Independent reference information is required to determine whether a particular change is real.

### 3.3.3. Sensitivity analysis

We assessed the sensitivity of the harmonised estimates to the spatial regularisation used to construct the baseline temporal-deviation field. The analysis considered nine Gaussian-kernel specifications obtained by combining three kernel sizes ( 3×3 , 7×7 , and 11×11 grid cells) with three Gaussian bandwidths ( σ=0.5,1.0,1.5 ). The forward and backward estimates were combined using the same time-varying blending weights as the main harmonisation procedure, thereby varying only the Gaussian spatial-kernel specification across sensitivity runs.

The analysis was conducted for Brazil, Nigeria, and Thailand, representing three different world regions, and separately for building count, building surface area, and building volume. For every parameter configuration, we calculated the normalised net change between 2016 and 2023:

$$G_{i}=\frac{X_{i,2023}-X_{i,2016}}{\max\left(X_{i,2016},X_{i,2023},1\right)}$$ (8)

where $X_{i,t}$ is the harmonised value for grid cell i in year t . This bounded measure ranges from -1 to 1 and permits comparison among metrics with different units and magnitudes.

### 3.4. Computational implementation and output generation

All processing was implemented in R using the terra package for raster operations. Parallel execution was enabled through the future and future.apply libraries ( Bengtsson, 2025), allowing country–metric combinations to be processed independently. For each ISO3 country and each building metric (count, surface area, and volume), the corresponding annual GeoTIFF files for the period 2016–2023 from Priyatikanto et al. (2025) were loaded as a multi-layer raster stack. Prior to processing, all layers were checked to ensure identical spatial extent, resolution, and coordinate reference system. The workflow described above was then applied to each raster time series. Processing was performed independently for each country and metric, enabling scalable production across the full dataset. The harmonised results were saved as single-band GeoTIFFs, one per country, metric, and year, retaining the input data’s spatial properties.

## 4. Results

### 4.1. Temporal-consistency assessment across countries

To evaluate the impact of the spatio-temporal smoothing procedure, we compared the raw and harmonised building-characteristics datasets using a set of diagnostics that quantify temporal consistency and the preservation of spatial patterns across the 130 Global South countries included in the dataset. Validation statistics were computed for each country and for three metrics: building surface area, building volume, and building count.

**4.1.1. Handling of temporal gaps**

**Figure 3.**
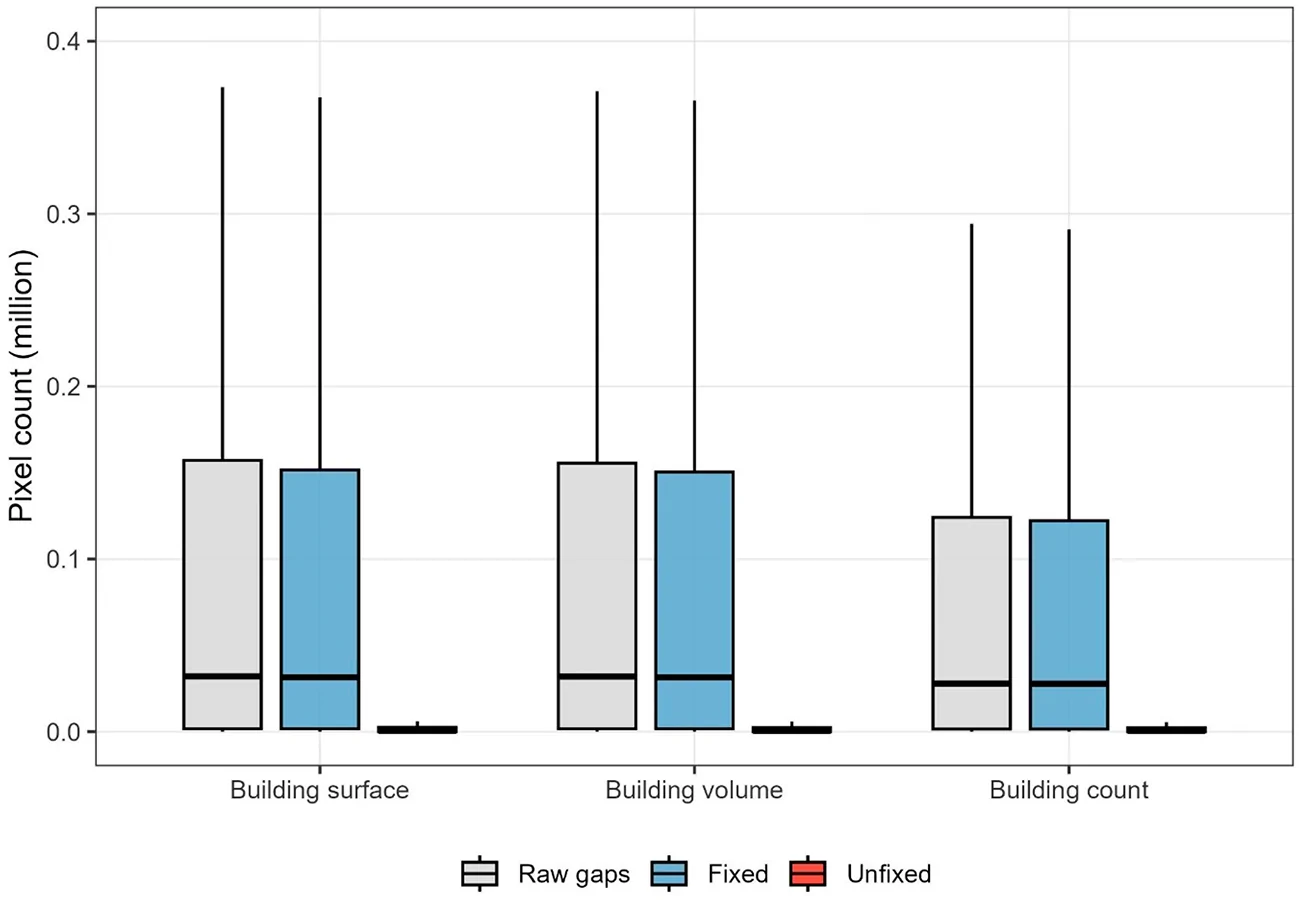
Country-level summary of temporal NA-gap handling for building surface area, building volume, and building count across the 130 Global South countries included in the dataset. Boxplots summarise the distribution of these country-level counts across countries. The central line indicates the median, the box shows the interquartile range, whiskers extend to 1.5 times the interquartile range, and points indicate outlying countries.

Because missing annual observations can also reduce temporal consistency, we quantified the number of pixels containing temporal gaps in the raw series, the number of those gaps resolved after smoothing, and the number remaining unresolved in Figure 3. These diagnostics were computed separately for each country and metric. For most countries and metrics, the number of resolved gaps closely matches the number of raw gaps, while the number of unresolved gaps is generally close to zero. This indicates that the smoothing workflow reconstructs most temporal gaps effectively without leaving substantial residual incompleteness.

**4.1.2. Reduction of temporal oscillations**

Spurious fluctuations in annual building metrics often appear as alternating increases and decreases between consecutive years. To quantify this behaviour, we identified sign-flip events in the annual differences of each pixel time series. Specifically, for each grid cell i , annual differences were computed as d(i,t)=r(i,t+1)−r(i,t) for t=2016,…,2022 , and a sign flip occurs when consecutive differences change sign, i.e., d(i,t)·d(i,t+1)<0 . Then, sign-flip events were defined by whether at least one such oscillation occurred during the time series. The total number of pixels exhibiting sign flip events was finally counted separately for the raw and harmonised datasets. A reduction in the number of oscillating pixels after smoothing indicates that the harmonisation procedure suppresses implausible year-to-year fluctuations.

**Figure 4.**
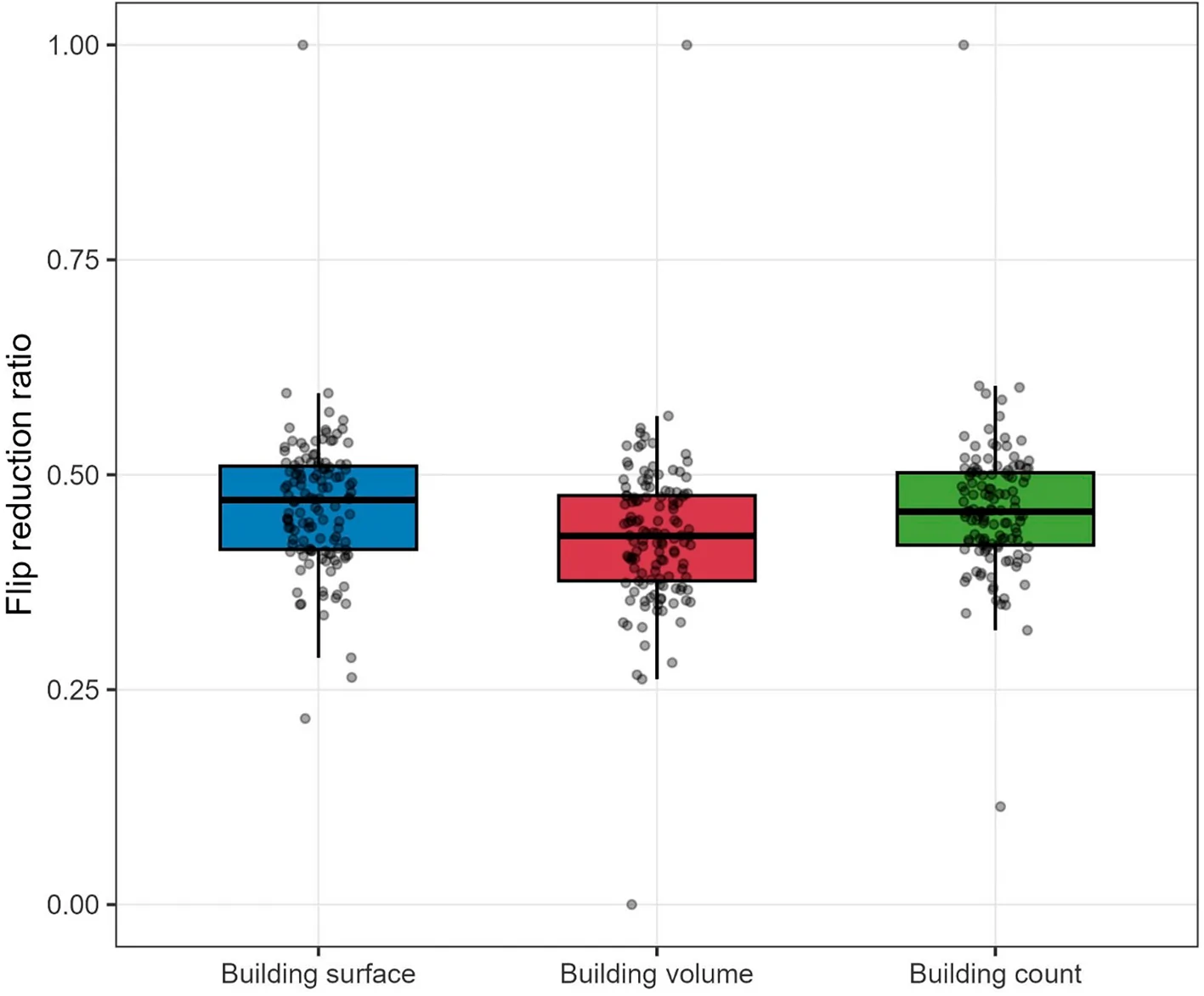
Country-level reduction in pixels exhibiting sign-flip behaviour after smoothing for building surface, building volume, and building count across the 130 Global South countries included in the dataset. Boxplots summarise the country distributions and points indicate individual countries.

Across countries, the number of pixels showing sign-flip behaviour decreased consistently after smoothing ( Figure 4). Median flip-reduction ratios were generally around 0.4, indicating that the harmonisation procedure removed a substantial share of implausible year-to-year oscillations, although the magnitude of reduction varied by metric and was lower for building volume than for building surface area and building count. This level of reduction is broadly consistent with Priyatikanto et al. (2025), who reported that polynomial temporal smoothing reduced fluctuating pixels by approximately 50%. The inter-country distributions indicate that the reduction was widespread rather than driven by a small number of outlying countries, suggesting that the harmonisation procedure systematically suppresses spurious temporal fluctuations while retaining broad spatial and temporal patterns.

**4.1.3. Zigzag amplitude metric**

In addition to counting oscillation events, we quantified the magnitude of oscillatory behaviour using a relative zigzag amplitude metric $Z_{\mathrm{rel}}$ . For each pair of consecutive annual differences, the oscillation amplitude was defined as $Z_{\mathrm{rel}}\left(i,t\right)=\min\left(|d\left(i,t\right)|,|d\left(i,t+1\right)|\right)/\max\left(r\left(i,t\right),r\left(i,t+1\right),r\left(i,t+2\right),\epsilon \right)$ , where ϵ is a small constant introduced to avoid division by zero. For each grid cell, the maximum $Z_{\mathrm{rel}}$ value across all years was retained as a summary of the strongest oscillation in the time series. Lower values in the harmonised dataset indicate attenuation of abrupt fluctuations.

**Figure 5.**
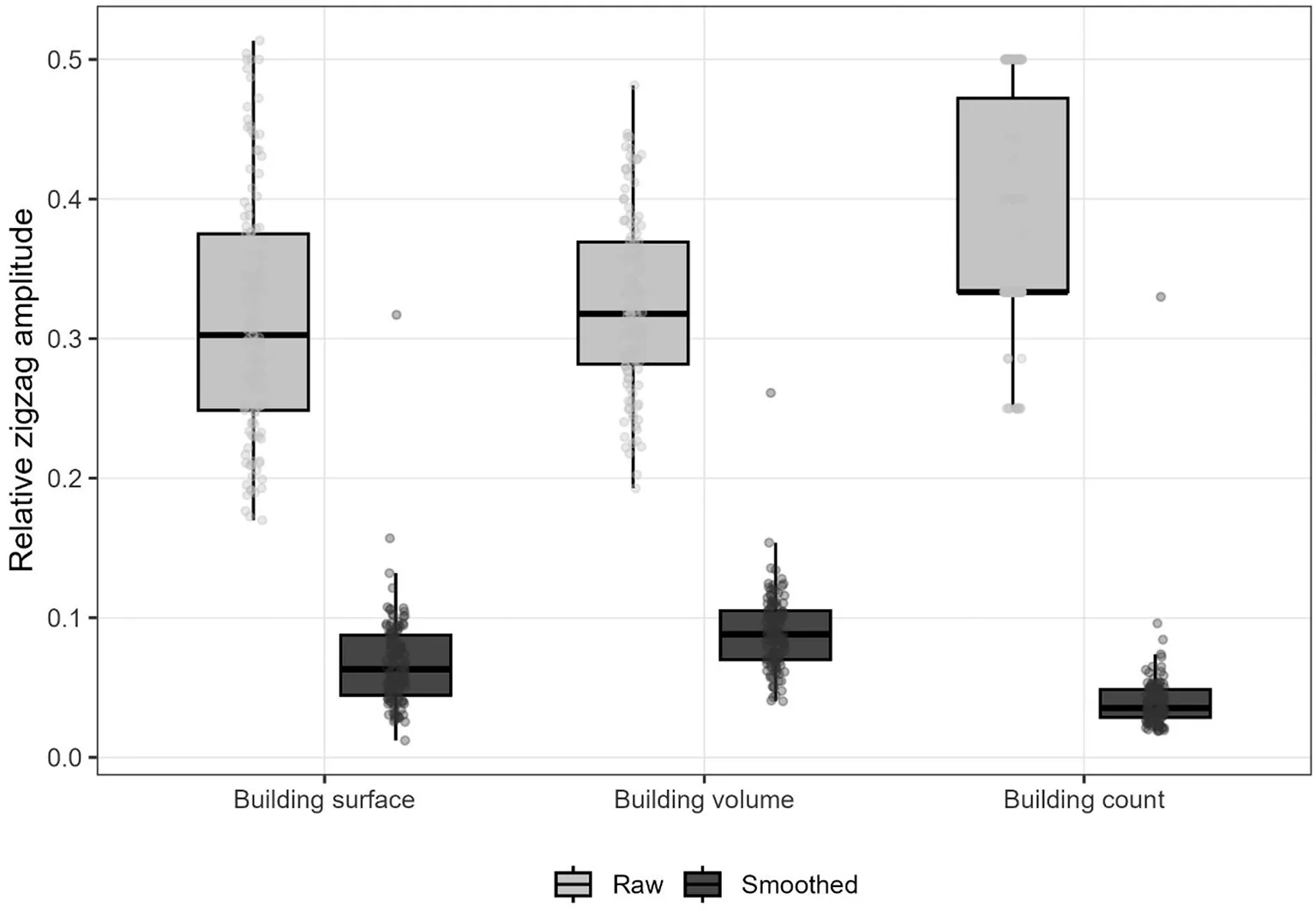
Country-level comparison of relative zigzag amplitude before and after temporal smoothing for building surface, building volume, and building count across the 130 Global South countries included in the dataset. Boxplots summarise the country distributions and points indicate individual countries.

The harmonised dataset shows a pronounced downward shift in relative zigzag amplitude across all three metrics ( Figure 5). Raw series exhibit substantially higher oscillation amplitudes, whereas the smoothed series cluster at much lower values. This pattern indicates that the harmonisation procedure reduces not only the frequency of oscillations, but also their magnitude. Because such abrupt reversals are likely to reflect uncertainty or random error in the annual input data, rather than real short-term changes in the built environment, the reduction in $Z_{\mathrm{rel}}$ suggests improved temporal precision and consistency of the harmonised product. However, this should not be interpreted as direct evidence of improved absolute accuracy, as independent ground-truth building data are not available for systematic validation and any bias present in the underlying input data may persist after smoothing.

**4.1.4. Preservation of spatial patterns**

**Figure 6.**
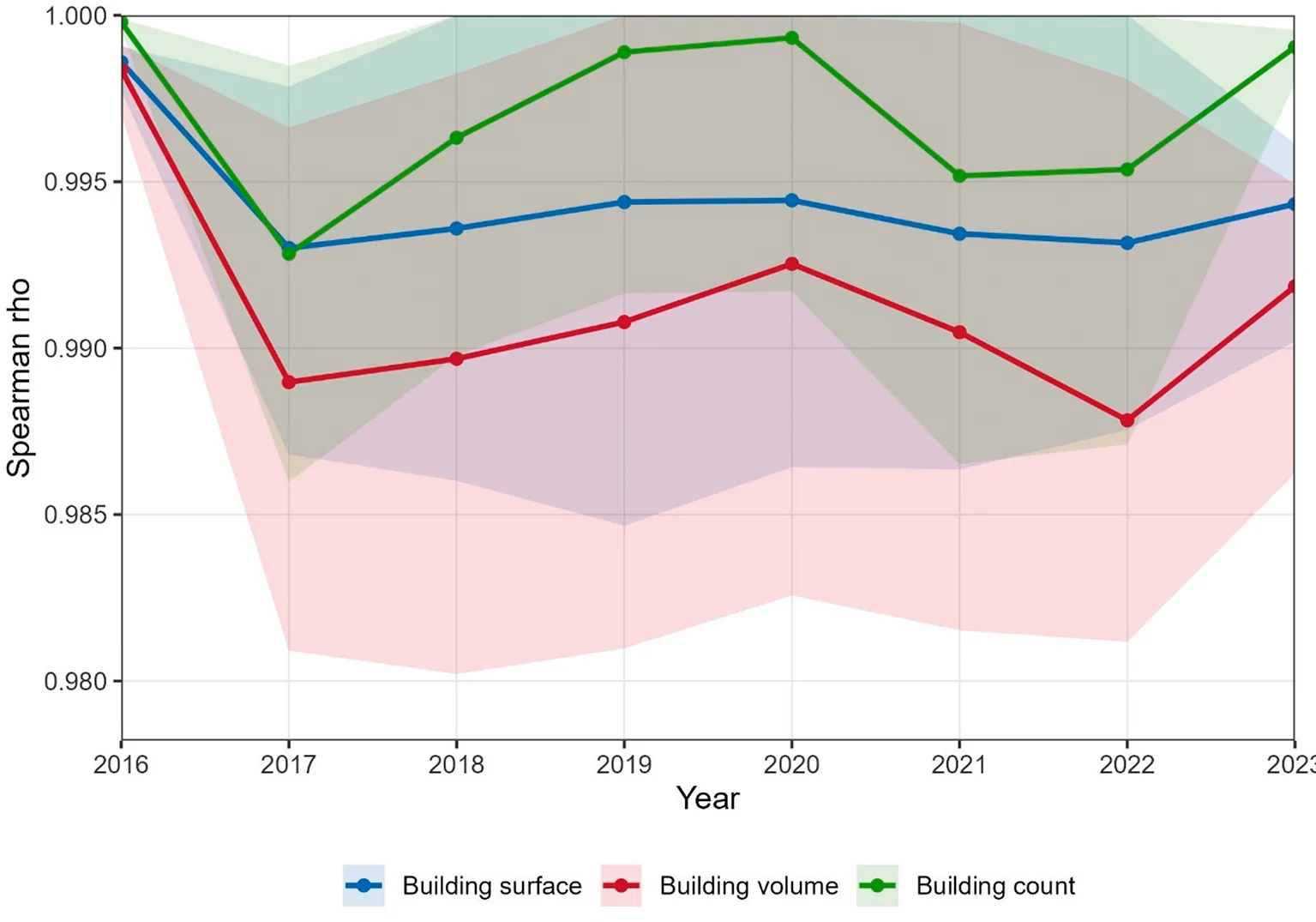
Annual Spearman rank correlation between raw and harmonised rasters across countries for building surface, building volume, and building count. Solid lines show the median correlation across countries and shaded ribbons indicate the interquartile range.

To verify that temporal smoothing does not substantially alter the spatial distribution of building characteristics, we computed Spearman rank correlations between the raw and harmonised rasters for each year. For each metric and year, the correlation was calculated across all valid grid cells within each country. Spearman correlations remain uniformly high throughout the covering period ( Figure 6). Median correlations across countries are consistently close to one for all metrics and years, and the interquartile ranges remain narrow. This indicates that the harmonisation procedure preserves the relative spatial ordering of grid-cell values while improving temporal consistency. In other words, smoothing reduces spurious temporal fluctuations without materially changing the spatial pattern of building surface, volume, or count.

### 4.2. City-level examples and interpretation of building metrics

Following the country-level validation across the 130 Global South countries, we use three city-level examples to illustrate how the harmonisation procedure behaves in contrasting local settings. Piracicaba, Brazil; Benin City, Nigeria; and Buri Ram, Thailand were selected as diagnostic case studies because they represent different combinations of temporal artefacts and settlement dynamics observed in the raw gridded layers. Piracicaba illustrates a relatively stable urban setting with limited asymmetric capping but clear smoothing eligibility across the consolidated urban footprint. Benin City represents a rapidly expanding urban area where stronger capping and smoothing effects are required to stabilise substantial temporal variability while preserving the signal of urban growth. Buri Ram provides an example of a smaller and more fragmented urban settlement, where temporal variability is present but capping affects only a limited subset of grid cells. These examples are not intended to be statistically representative of all cities in the dataset; rather, they provide interpretable case studies showing how the method handles intermittent missing observations, abrupt annual gains or losses, and short-term oscillations while retaining broad spatial and temporal structure.

The three metrics provide complementary perspectives on settlement change and should be selected according to the research objective. Building count is most informative for examining changes in the number and spatial distribution of structures, although it should not be interpreted directly as a dwelling or household count. Total building footprint area captures horizontal land occupation and is therefore particularly suitable for studying settlement expansion, land consumption, and changes in built-up extent. Building volume incorporates estimated height and provides an indicator of vertical development and built mass, making it useful for investigating densification, approximate floor-space capacity, population distribution, energy demand, and urban microclimate. No single metric is universally preferable, and joint interpretation can distinguish different development processes. For example, increasing footprint area without a comparable increase in building count may indicate enlargement or consolidation of existing structures, whereas increasing volume relative to footprint area may indicate vertical densification. Building volume should nevertheless be treated as a proxy rather than a direct measure of usable floor space because it does not capture internal floor configuration or building function.

**4.2.1. Data preprocessing and temporal consistency diagnostics**

**Figure 7.**
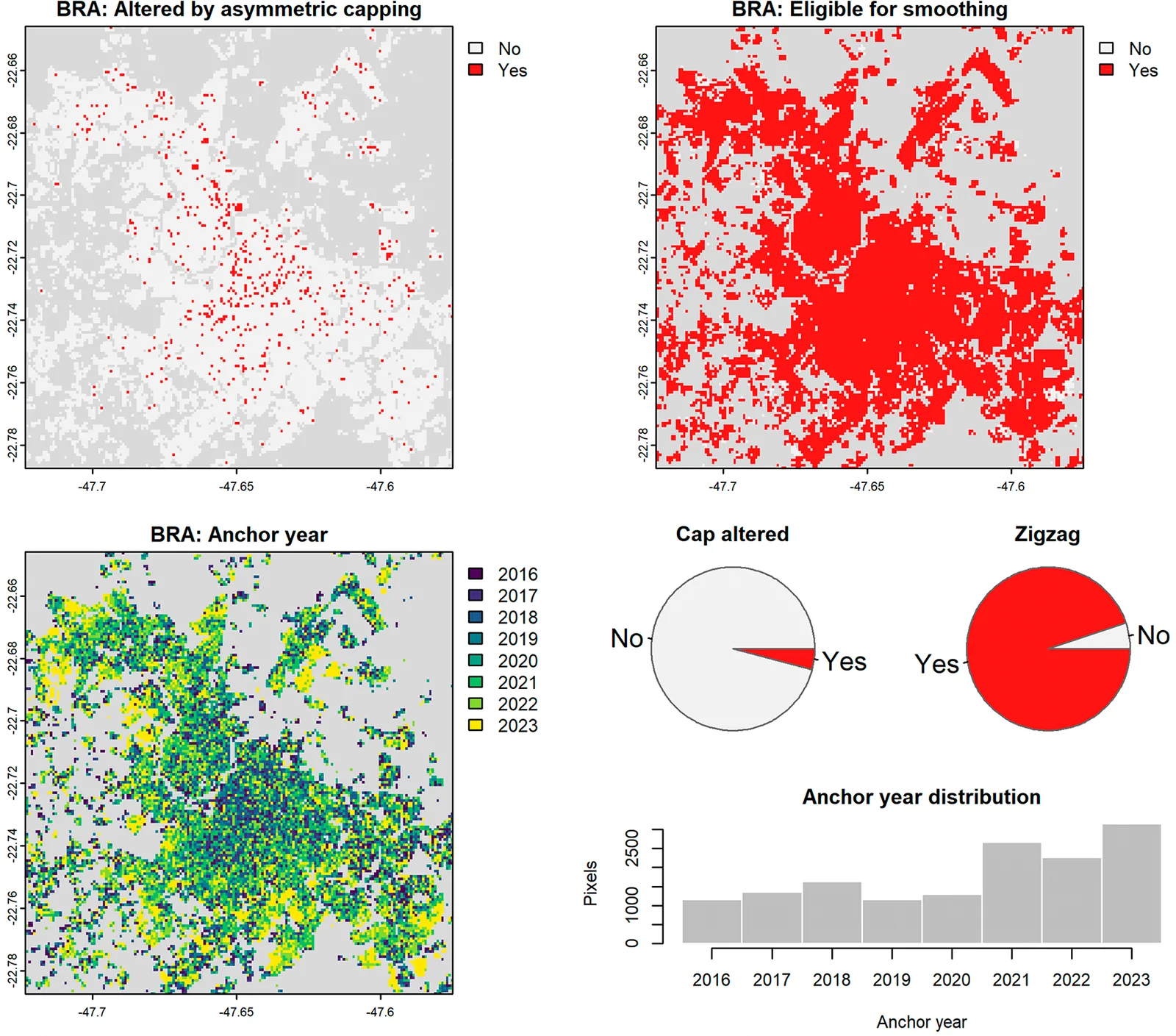
Temporal smoothing diagnostics and anchor year distribution in Piracicaba, Brazil.

The three city examples show contrasting preprocessing and smoothing behaviour across different settlement contexts. In Piracicaba, Brazil, asymmetric capping affects only a small and spatially dispersed subset of grid cells, indicating that large implausible annual jumps are relatively limited in this example ( Figure 7). However, many grid cells within the consolidated urban footprint are eligible for temporal smoothing, suggesting that the main issue is not extreme outliers but smaller year-to-year oscillations in the raw time series. The anchor-year distribution is relatively balanced across the study period, with only a slight shift towards more recent years, consistent with a comparatively stable urban setting in which smoothing mainly improves temporal coherence while introducing only limited preprocessing adjustments.

**Figure 8.**
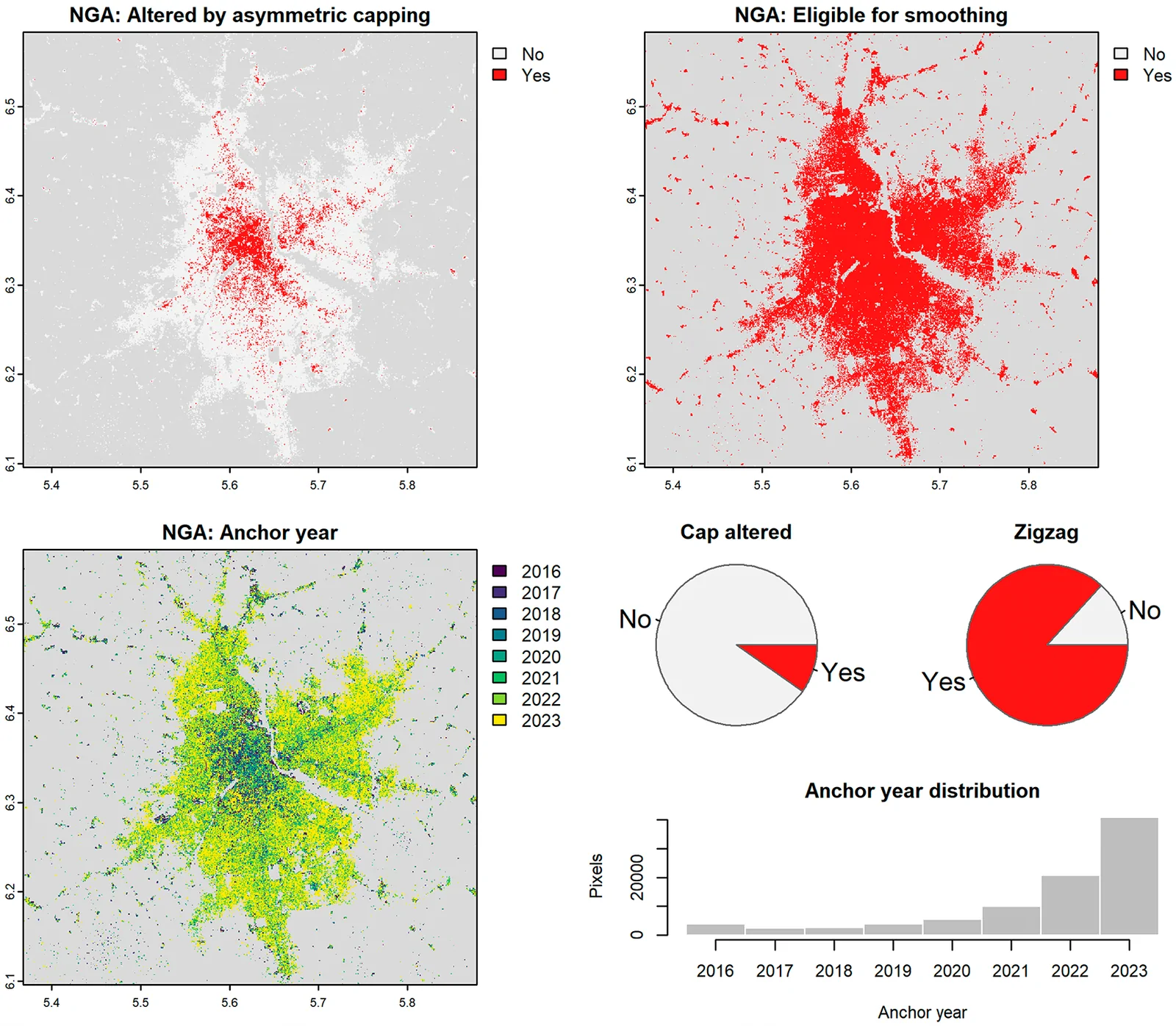
Temporal smoothing diagnostics and anchor year distribution in Benin City, Nigeria.

Benin City, Nigeria, shows a different pattern as shown in Figure 8. Here, asymmetric capping affects a larger share of grid cells and is more spatially concentrated within the urban core and along expansion corridors. Almost the full mapped urban extent is eligible for temporal smoothing, and the anchor-year distribution is strongly skewed towards later years. Together, these diagnostics indicate a more dynamic and temporally variable setting, where the raw annual series contain both genuine expansion signals and stronger short-term fluctuations. In this case, the preprocessing and smoothing steps are more active, helping to stabilise the time series while retaining the broader pattern of recent urban growth.

**Figure 9.**
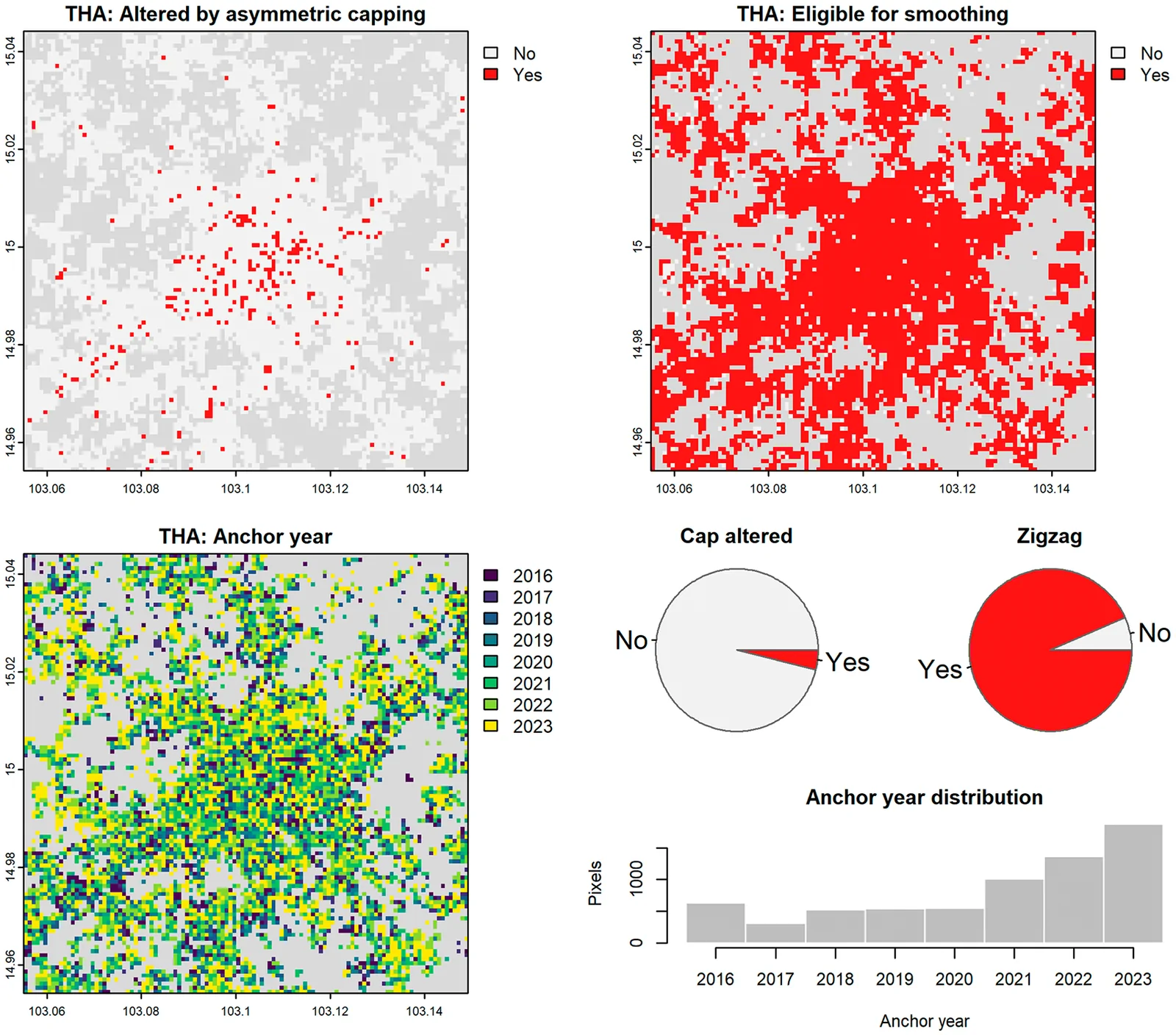
Temporal smoothing diagnostics and anchor year distribution in Buri Ram, Thailand.

Figure 9 further shows that Buri Ram, Thailand, provides an intermediate but more spatially fragmented example. As in Piracicaba, asymmetric capping affects only a limited number of grid cells, suggesting that extreme annual changes are uncommon. However, smoothing eligibility remains widespread across the mapped area, and the anchor years are heterogeneous with a moderate concentration in recent years. This pattern indicates that the raw data contain widespread but generally lower-magnitude temporal variability rather than strongly localised outliers. The harmonisation therefore mainly acts to reduce short-term oscillations across a fragmented settlement structure, with relatively little reliance on asymmetric capping.

**4.2.2. Smoothing effect**

**Figure 10.**
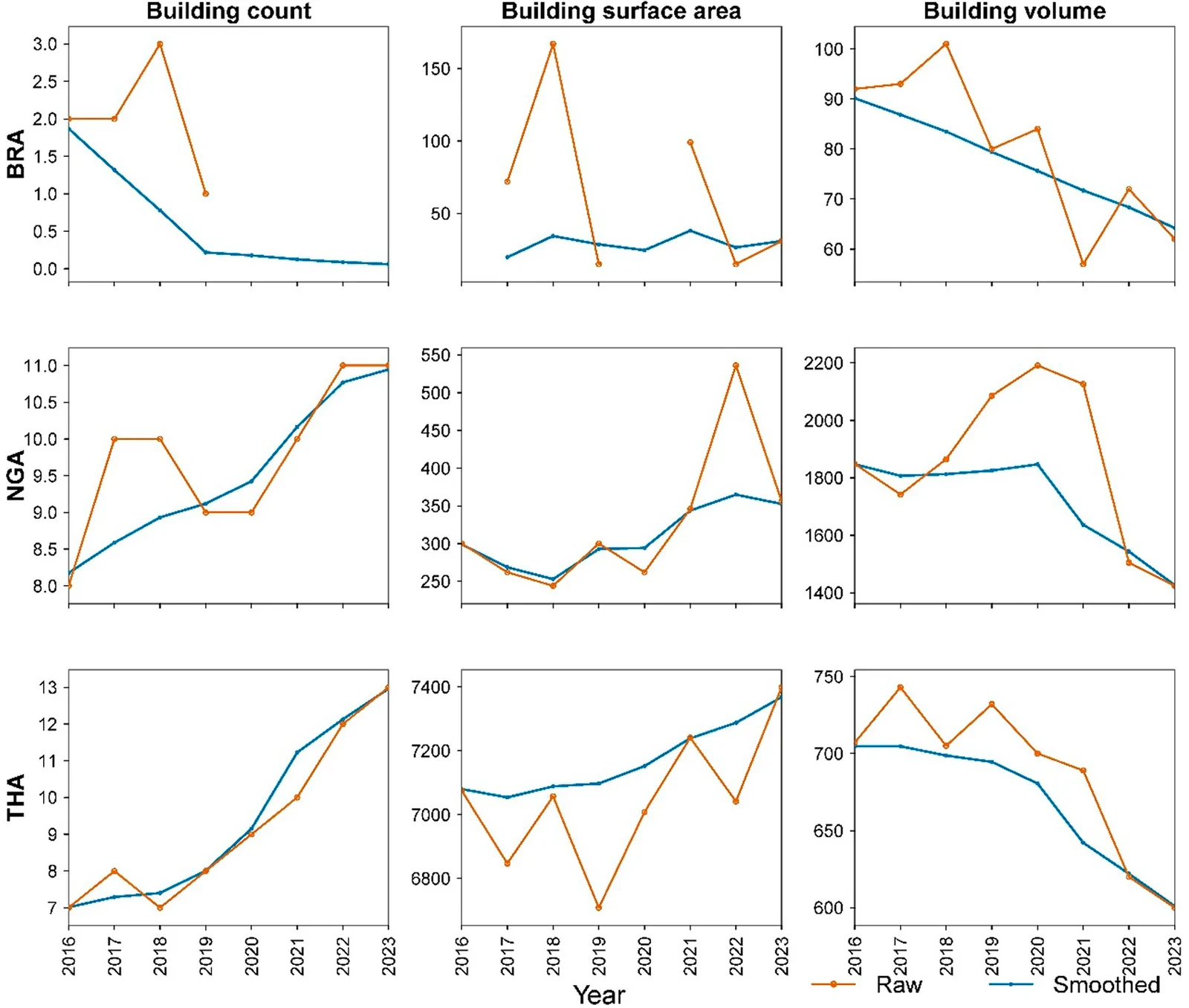
Illustrative temporal trajectories of raw and smoothed building metrics for a pixel in each country and metric combination.

Figure 10 presents selected grid-cell trajectories from the three city examples to show typical local effects of the harmonisation procedure on building count, surface area, and volume. The grid cells were chosen from locations where the raw annual series displayed common temporal artefacts identified in the diagnostics, including isolated spikes, abrupt reversals, missing intermediate observations, or short-term oscillations. They are therefore illustrative examples rather than a random sample of all grid cells. In general, the harmonised series generally preserves the longer-term temporal signal in the raw data while reducing short-term reversals and abrupt fluctuations.

For building count, the smoothed trajectories are more stable than the raw series while retaining the main direction of change. In Brazil, the raw series shows abrupt variations early in the record, whereas the harmonised series follows a steadier decline towards near-zero values. In Nigeria and Thailand, the smoothed count series preserves the general upward tendency of the raw data but reduces alternating year-to-year increases and decreases. These examples show that the method can dampen zigzag behaviour while retaining broader changes in building presence or density.

For building surface area, the smoothing effect is particularly clear. In Brazil, the raw series contains strong fluctuations and also includes missing intermediate years between valid observations. These gaps are likely related to temporary limitations in the source satellite imagery, for example cloud cover, haze, or locally poor image quality, rather than true disappearance of built-up area. The harmonised series bridges these missing years in a temporally consistent way using information from surrounding valid observations, producing a continuous trajectory that is more plausible than the fragmented raw record. Similar damping of short-term volatility is visible for Nigeria and Thailand, where peaks and dips in the raw series are moderated while the overall level and direction of change are retained.

For building volume, the harmonised series again reduces abrupt fluctuations but maintains the broader temporal structure. In Brazil, the smoothed curve converts a sequence of alternating increases and decreases into a more gradual decline. In Nigeria, the raw series shows pronounced short-term peaks followed by a strong drop, whereas the harmonised trajectory remains smoother and more internally consistent. In Thailand, the smoothed volume series follows the general downward pattern of the raw data but suppresses intermediate oscillations. This indicates that the procedure is able to stabilise volume estimates without removing meaningful longer-term variation.

**4.2.3. Pattern and dynamics of settlement growth**

**Figure 11.**
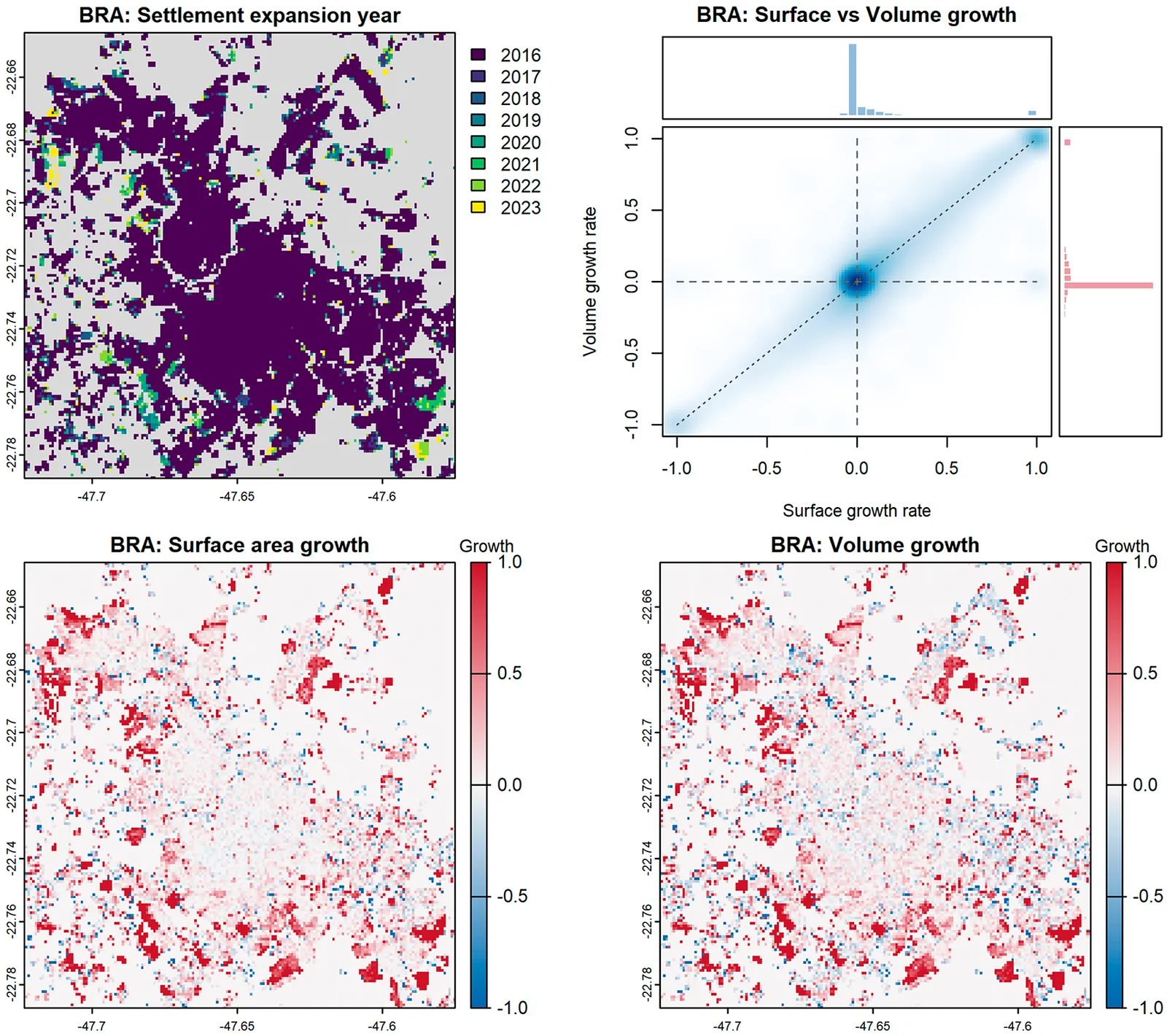
Spatial and statistical characterization of urban development in Piracicaba, Brazil.

The three city examples also illustrate how the harmonised dataset can be used to examine spatial patterns of building-stock change while distinguishing between early-detected built-up areas and later-emerging grid cells. In Piracicaba, Brazil, the first valid year map shows a consolidated urban core dominated by early observations, with later detections occurring mainly along the urban fringe and in scattered infill locations ( Figure 11, top-left). The relationship between surface-area growth and volume growth is tightly clustered around the diagonal, indicating close coupling between horizontal and volumetric change ( Figure 11, top-right). The blended growth maps show coherent areas of positive change around the urban edge, while the central built-up area remains comparatively stable ( Figure 11, bottom panels). This pattern is consistent with incremental outward expansion and localised densification, although the example is intended primarily as an illustration of dataset behaviour rather than a definitive assessment of urban development processes.

**Figure 12.**
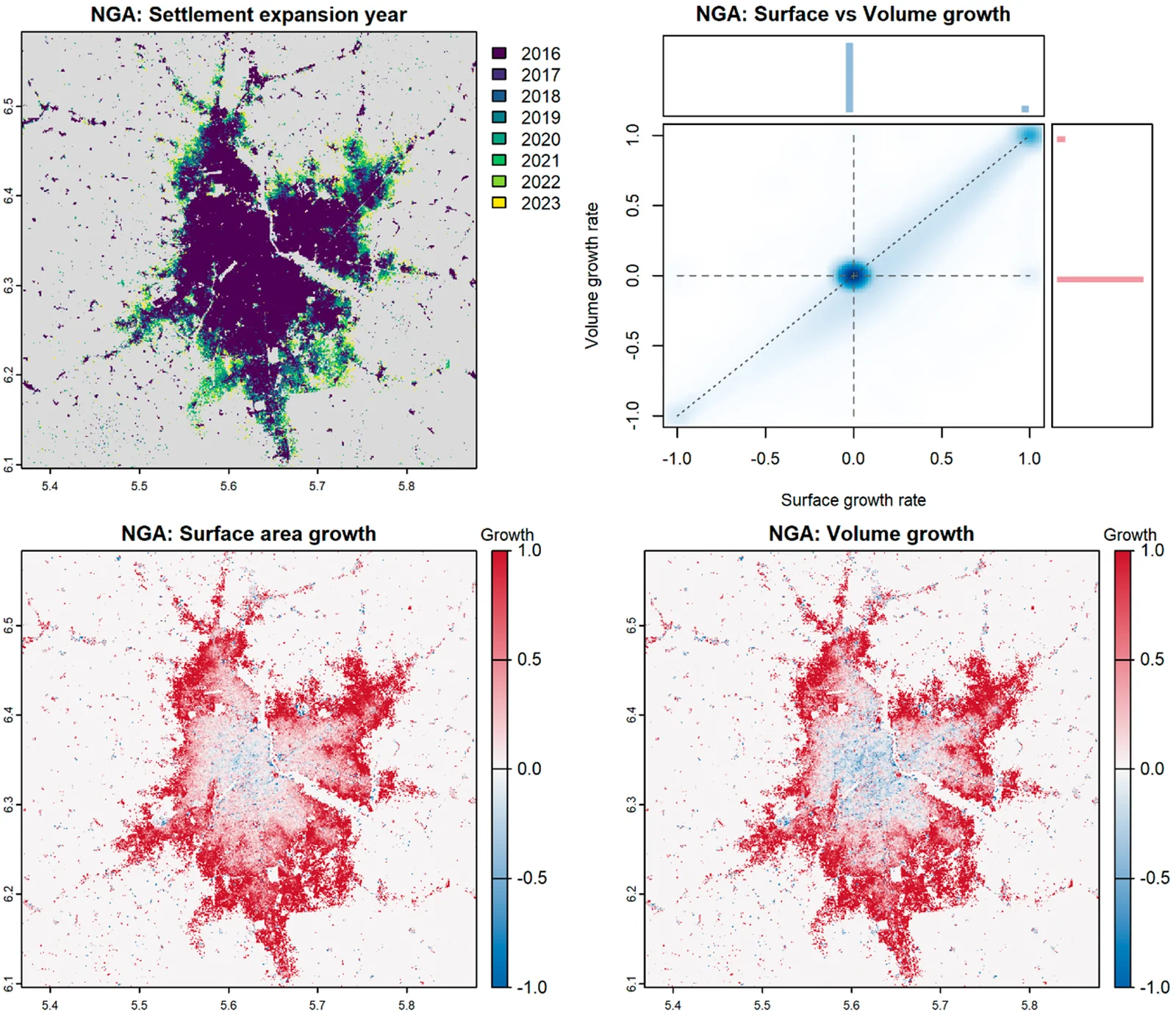
Spatial and statistical characterization of urban development in Benin City, Nigeria.

Benin City, Nigeria, shows a stronger and more spatially extensive growth signal. The first valid year map indicates an early-detected urban core, with later observations extending outward along peripheral corridors and surrounding expansion areas ( Figure 12, top-left). Surface-area and volume growth are positively associated, with many grid cells showing simultaneous increases in both metrics ( Figure 12, top-right). Spatially, surface-area growth is widespread around the urban fringe, whereas volume growth is more heterogeneous, including areas of weak or negative change within parts of the core ( Figure 12, bottom panels). These patterns are consistent with rapid outward settlement expansion accompanied by more variable changes in building volume, demonstrating how the harmonised product can capture both horizontal growth and changes in the vertical dimension of building stock.

**Figure 13.**
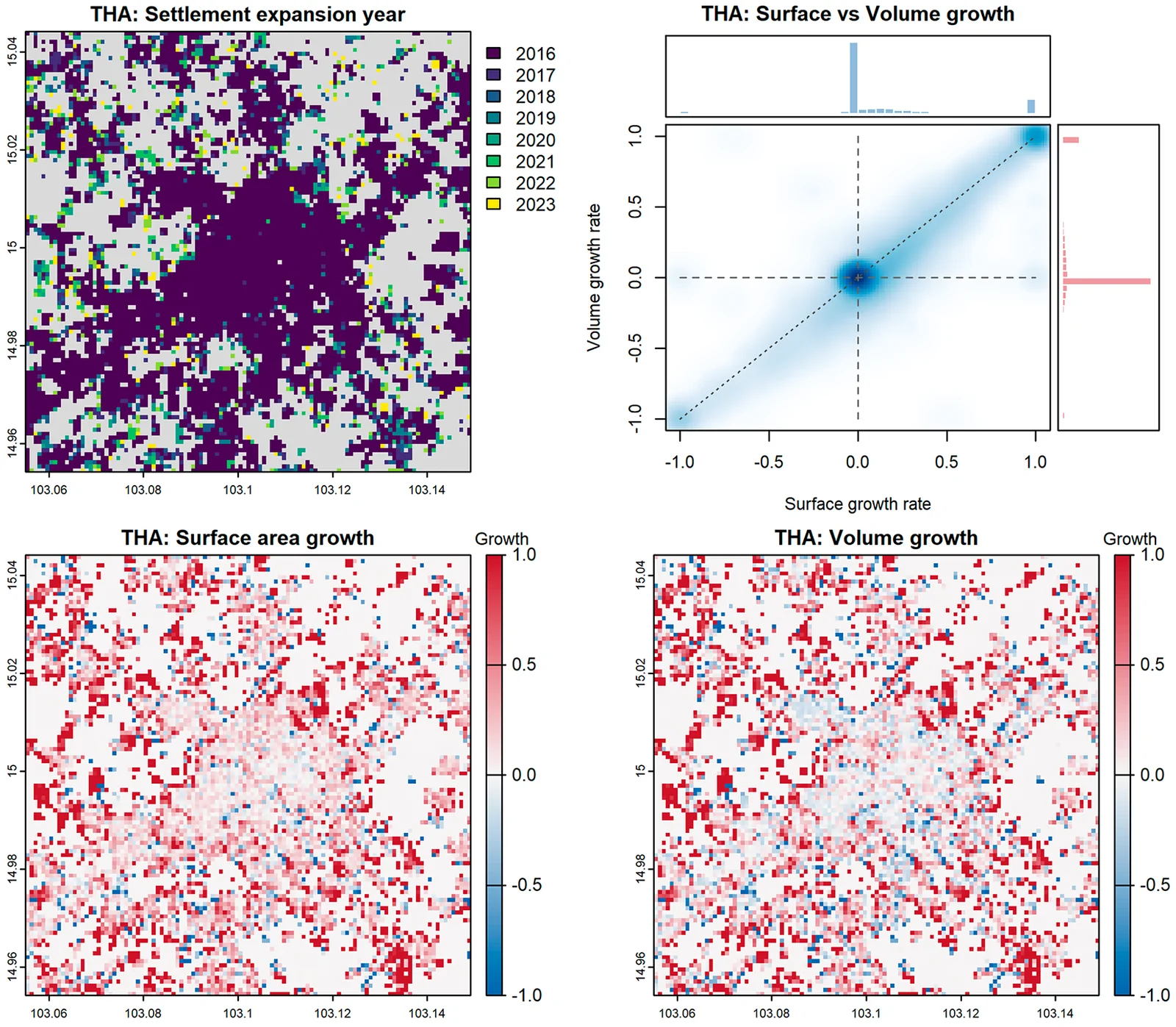
Spatial and statistical characterization of urban development in Buri Ram, Thailand.

Buri Ram, Thailand, presents a more fragmented and lower-magnitude pattern of change. Early valid observations form a compact but discontinuous built-up area, with later detections distributed as smaller peripheral and infill patches ( Figure 13, top-left). The relationship between surface-area and volume growth remains positive but is more diffuse than in the other two examples, suggesting weaker coupling between horizontal expansion and volumetric change ( Figure 13, top-right). The blended growth maps show patchy and relatively low-magnitude changes in both surface area and volume ( Figure 13, bottom panels). This example illustrates how the harmonised dataset represents more gradual and spatially dispersed settlement change in a smaller urban setting.

### 4.3. Sensitivity analysis

To evaluate whether the estimated 2016-2023 net building change was sensitive to the Gaussian spatial-kernel specification, we repeated the analysis across nine combinations of kernel size ( k=3,7, and 11 ) and Gaussian bandwidth ( σ=0.5,1.0, and 1.5 ) for the three AOIs and all three metrics: building count, surface area, and volume. Results were compared against the reference setting ( k=3,σ=0.8 ).

**Figure 14.**
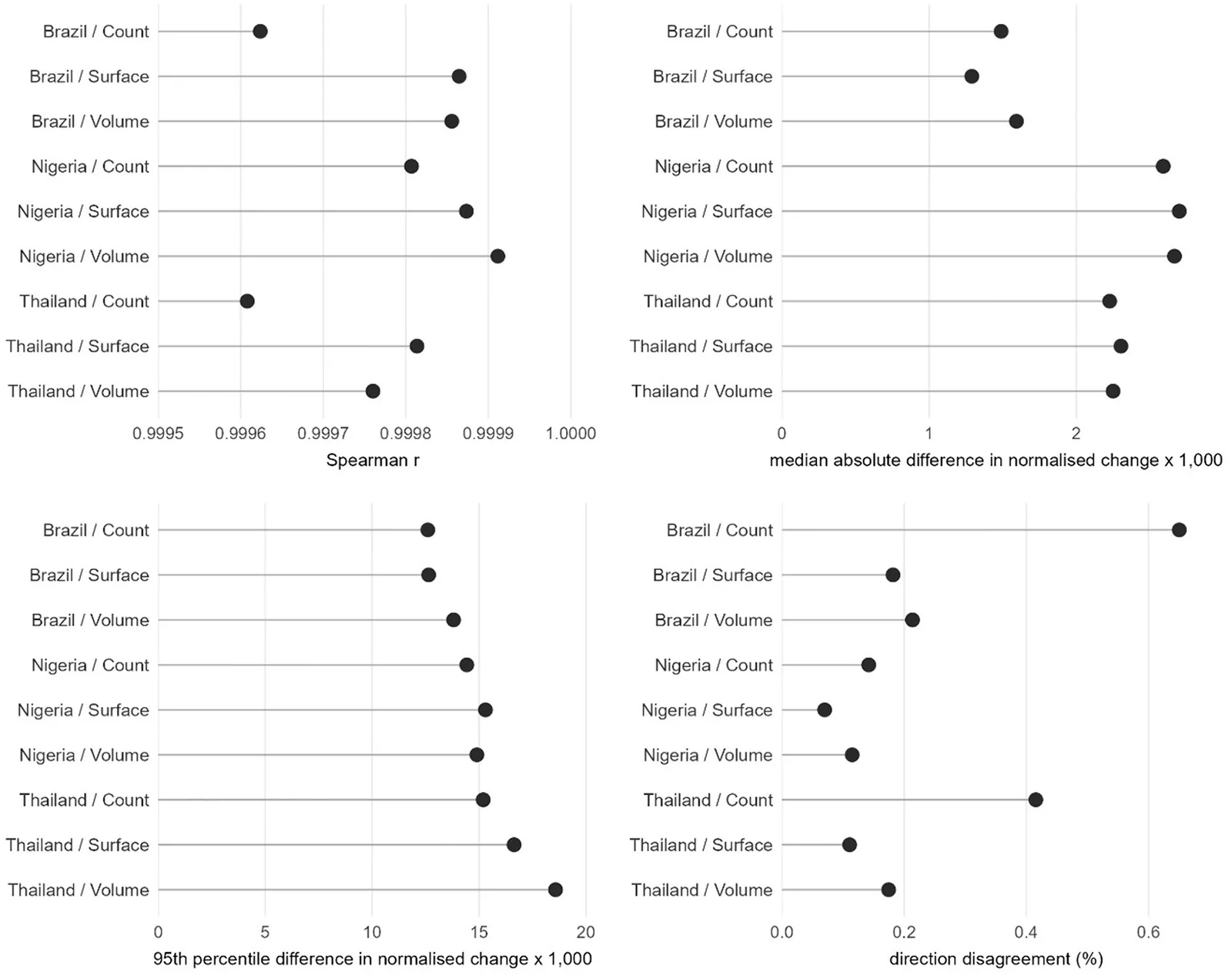
Sensitivity of normalised 2016-2023 net building change to Gaussian kernel settings.

The normalised net-change maps were highly stable across kernel settings ( Figure 14). Across all AOI-metric combinations, Spearman rank correlations with the reference setting were consistently very high ( ρ = 0.9996-0.9999). Median absolute differences in normalised net change were small, ranging from 0.0002 to 0.0027, and the 95th percentile absolute difference remained below 0.019 in all cases. Directional disagreement, defined as cells switching between positive, negative, or no net change relative to the reference setting, was also low, with a maximum of 0.65% across all comparisons.

Points show the worst value across eight non-reference kernel alternatives for each AOI-metric combination, using k = 3, σ = 0.8 as the reference setting. Lollipop lines extend from the axis baseline to the observed value. Higher Spearman ρ and lower absolute differences or direction disagreement indicate greater robustness.

Sensitivity was slightly greater in the Thai AOI, particularly for volume, where the largest 95th percentile absolute difference was 0.0186 under k = 11, σ = 1.5. However, even in this worst-case comparison, rank agreement remained extremely high ( ρ = 0.99976) and directional disagreement was only 0.17%. These results indicate that the spatial pattern and direction of estimated net building change are robust to plausible variation in the Gaussian smoothing kernel.

## 5. Discussions and conclusions

This study presents a spatio-temporally harmonised gridded building-characteristics dataset ( Zhang et al., 2026) for 130 countries in the Global South covering the period 2016–2023 at a spatial resolution of 3 arc-seconds, approximately 100 m at the equator. The dataset provides annual estimates of three complementary building metrics: building count, total building footprint area, and estimated building volume. These metrics capture different dimensions of building stock, including the number of structures, horizontal built-up extent, and the vertical dimension of the built environment.

The harmonised dataset is intended to support analyses requiring temporally consistent representations of the built environment at regional, national, and multi-country scales. By reducing implausible year-to-year fluctuations while preserving broad spatial patterns and multi-year trajectories, the dataset is particularly suited to applications such as settlement expansion monitoring, urbanisation analysis, exposure modelling, infrastructure assessment, and use as a covariate in gridded population models. Previous studies ( Tatem, 2017; Rubinyi et al., 2021; Nieves et al., 2021; Boo et al., 2022; Lochhead et al., 2026) have shown that building-footprint and settlement data can substantially improve population mapping, exposure assessment, and analyses of the built environment, particularly where conventional census or administrative data are sparse or infrequently updated.

The temporal-consistency assessment indicates that the harmonisation procedure substantially reduces irregular year-to-year variation. Across countries and metrics, smoothing reduced sign-flip behaviour and relative zigzag amplitudes, suggesting a reduction in short-term random fluctuations in the annual input data. This is consistent with previous work showing that satellite-derived time series can contain artefacts caused by variation in observation conditions, image availability, preprocessing, and model performance, and that temporal smoothing can reduce such noise while preserving broader trends ( Chen et al., 2004; Jönsson and Eklundh, 2004; Gómez et al., 2016; Qiu et al., 2019; Frantz et al., 2023). At the same time, high Spearman correlations between the raw and harmonised gridded layers indicate that the procedure preserves the relative spatial distribution of building characteristics. However, these diagnostics demonstrate improved temporal consistency rather than improved absolute accuracy. The harmonised product should therefore be interpreted as a temporally stabilised representation of the source building detections, rather than as independently validated estimates of true building-stock change.

Several limitations should be considered when using the dataset. The harmonisation algorithm is designed to suppress isolated spikes, drops, and short-term oscillations that are likely to reflect detection noise, incomplete observations, or variation in image availability and model performance. Although this improves temporal precision and consistency, the procedure cannot definitively distinguish observation artefacts from genuine changes in building stock. Consequently, it may attenuate genuinely abrupt changes occurring within a single year, particularly in locations experiencing rapid construction, demolition, redevelopment, or disaster-related damage. The dataset is therefore best suited to analysing multi-year trends and spatial patterns rather than detecting short-term individual events.

The evaluation presented here does not constitute independent validation against true annual building-stock change. Such validation was constrained by the limited availability of temporally consistent reference data for building count, footprint area, and volume at comparable spatial and annual resolutions across the study regions. Potential reference sources, including cadastral records, local building-footprint datasets, OpenStreetMap histories, and manually interpreted high-resolution imagery, often differ in temporal coverage, completeness, building definitions, and update practices.

The harmonised dataset uncertainties from the underlying Google Open Buildings 2.5D Temporal dataset and the gridded building-characteristics product from which it is derived ( Sirko et al., 2023; Priyatikanto et al., 2025). Detection performance may vary across regions owing to differences in satellite image quality, settlement morphology, roof materials, vegetation cover, and building density, as has been documented for building-footprint and built-up area products more generally ( Kraff et al., 2020; Chamberlain et al., 2024; Minghini et al., 2024). Temporal smoothing can reduce irregular variation but cannot remove systematic errors in the source detections.

Users should exercise particular caution when interpreting individual grid cells, isolated annual changes, sparsely built rural areas, locations with substantial missing observations, and areas undergoing abrupt physical change. Reliability is expected to be greater for aggregate spatial patterns and persistent multi-year trajectories than for individual buildings or single-year changes. For detailed local applications, the harmonised estimates should be evaluated alongside higher-resolution imagery, local cadastral information, independently produced building footprints, field observations, or other reference sources where available. Independent evaluation across dense urban, peri-urban, and rural contexts remains an important priority for future work.

Overall, the dataset provides a temporally consistent multi-year representation of building characteristics across data-scarce regions of the Global South. By combining annual gridded building metrics with a reproducible spatio-temporal harmonisation workflow, it offers a useful resource for research and policy applications requiring temporally coherent information on settlement change, infrastructure distribution, population exposure, and built-environment dynamics. Its principal value lies in supporting aggregate spatial analyses and multi-year comparisons; it should not be interpreted as an independently validated record of individual building changes or short-term events.

## Data Availability

The source building-characteristics product used as input to this study is publicly available from the University of Southampton/WorldPop Open Data Repository: High spatial resolution building characteristics for the Global South: insights from the Google Open Buildings Temporal Dataset, 2016–2023. https://doi.org/10.5258/SOTON/WP00850. This source dataset contains annual gridded building-characteristics layers derived from the Google Open Buildings 2.5D Temporal dataset and aggregated to the WorldPop master grid at a spatial resolution of 3 arc-seconds, approximately 100 m at the equator. Repository: A temporally harmonised gridded building-stock dataset for the Global South, 2016–2023, version 1.0. https://dx.doi.org/10.5258/SOTON/WP00887. The data can also be accessed via the WorldPop data repository at: https://data.worldpop.org/repo/prj/Google_OBT_v2_5/v1_2_S/T_0_5/ This project contains the following underlying data:
• Annual harmonised building count GeoTIFF files for 130 countries in the Global South, 2016–2023. • Annual harmonised total building surface area GeoTIFF files for 130 countries in the Global South, 2016–2023. • Annual harmonised estimated building volume GeoTIFF files for 130 countries in the Global South, 2016–2023. Files are organised by country, metric, and year. Each file is distributed as a single-band GeoTIFF aligned with the WorldPop master grid at a spatial resolution of 3 arc-seconds, approximately 100 m at the equator, in EPSG:4326. The GeoTIFF files use a NoData value of −99999. Data are available under the terms of the Creative Commons Attribution 4.0 International licence. No additional extended data are associated with this article. Repository: R script for the smoothing procedure of high-resolution building-stock characteristics for the Global South, based on Google Open Buildings 2.5D Temporal Dataset, 2016–2023, version 1.0. Zenodo. https://doi.org/10.5281/zenodo.19948835. The archived software contains the R scripts used to implement the temporal gap handling, asymmetric cap adjustment, Kalman-inspired forward–backward smoothing procedure, output generation, and technical validation diagnostics described in this article. Software is available under the terms of the Creative Commons Attribution 4.0 International licence.
